# Weight Regain in the Second Year after Sleeve Gastrectomy Could Be a Predictor of Long-Term Outcomes?

**DOI:** 10.3390/medicina59040766

**Published:** 2023-04-15

**Authors:** Jan Kapała, Tomasz Maroszczuk, Julia Lewandowska, Paweł Lech, Natalia Dowgiałło-Gornowicz

**Affiliations:** Department of General, Minimally Invasive and Elderly Surgery, Collegium Medicum, University of Warmia and Mazury, Niepodległosci 44 St., 10-045 Olsztyn, Poland; jan.kapala@student.uwm.edu.pl (J.K.); tomasz.maroszczuk@student.uwm.edu.pl (T.M.); julia.lewandowska.1@student.uwm.edu.pl (J.L.); lechpawel@op.pl (P.L.)

**Keywords:** sleeve gastrectomy, weight regain, long-term follow-up, weight predictor, weight regain predictor

## Abstract

*Background and Objectives*: Sleeve gastrectomy (SG) is an effective surgical procedure in the treatment of obesity. However, a significant percentage of patients suffer from weight regain over long-term follow-up. The mechanisms responsible for this process are still poorly understood. The aim of the study is to evaluate the predictive effect of weight regain in the second year after SG on long-term bariatric surgery effectiveness. *Methods:* A retrospective cohort study was performed using the database of routinely collected information about patients undergoing SG in the Department of General, Minimally Invasive and Elderly Surgery in Olsztyn. Patients were divided into two groups according to the change in body weight between the first and second years after the surgery: weight gainers (WG) and weight maintainers (WM). *Results:* A study group consisting of 206 patients, with follow-up over 5 years, was included in the study. The WG group consisted of 69 patients while the WM group had 137 patients. There were no significant differences between the patient characteristics (*p* > 0.05). The WM group had a mean %EWL of 7.45% (SD, 15.83%) and %TWL of 3.74 (SD, 8.43). The WG group had a mean %EWL of 22.78% (SD, 17.11%) and %TWL of 11.29% (SD, 8.68). The difference between the groups was statistically significant (*p* < 0.05). The study showed significantly better results in WM compared to WG (*p* < 0.05). *Conclusion:* Weight regain in the second year after SG may be a good factor for long-term bariatric surgery effectiveness prognosis.

## 1. Introduction

Obesity is an abnormal, excessive accumulation of adipose tissue, posing a threat to human health. It is generally classified by body mass index (BMI) which is weight in kilograms divided by height in meters squared [1]. For adults, BMI between 25 and 29.9 kg/m² is defined as overweight and BMI above 30 kg/m² is defined as obesity.

According to a World Health Organization (WHO) report from 2016, the prevalence of overweightness among adults was 39% and one-third of them were obese. It also concerns children, which means that over 340 million children and adolescents aged 5–19 were overweight or obese in 2016 and about 39 million children under the age of 5 were overweight or obese in 2020 [2].

Obesity is basically caused by calories intake exceeding individual caloric demand. Pathogenesis seems to be multifactorial and is related to endogenous and exogenous factors [3,4].

Obesity affects almost every system in the human body, causing a substantial number of comorbidities. All the complications increase mortality and morbidity, causing a decrease in life expectancy. Moreover, relative to normal weight, obesity is associated with significantly higher all-cause mortality [5].

Obesity and its complications can be treated with bariatric surgery, which is the most effective method [6]. Compared to other obesity treatments, bariatrics stands out for its long-term weight loss [7]. Nowadays, it is performed laparoscopically, which makes the procedure less invasive and reduces the risk of postoperative complications.

Sleeve gastrectomy (SG) is the most common bariatric procedure [8]. The surgery involves removing about two-thirds of the stomach and creating a thin tube from the rest of it. Finally, the stomach’s resected part is removed from the body [9].

The weight loss of patients undergoing SG is related to mechanisms involving the digestive, endocrine, and nervous systems. The feeling of hunger is reduced, the feeling of satiety is increased, and the digestive system’s absorption is reduced. Thus, patients experience a substantial caloric deficit, resulting in weight loss in the post-surgery period.

In numerous studies, SG has been shown to provide a considerable weight loss in short-term follow-up. According to a systematic review gathering 5218 individuals, basal mean BMI equal to 43.8 (SD, 8) decreased after 12 months to 30.7 (SD, 3.9). Simultaneously, the prevalence of DM2, AH and dyslipidaemia dropped by 81.9%, 66.5%, and 64.1%, respectively [10]. However, in long-term follow-up, a significant number of patients suffer from weight regain. In a systematic review gathering in nine studies, 1369 individuals’ regain ranged from 5.7% at 2 years to 75.6% at 6 years. However, because of their indirectness, these data have a poor level of certainty, especially because articles define weight regain differently [11,12,13,14]. Mechanisms responsible for obesity recurrence in the postoperative period are still incompletely understood because of the dearth of comprehensive long-term data which holistically evaluate the weight regain factors. Surgical factors (bougie size, residual stomach volume, gastric dilatation), hormonal and metabolic imbalance, behavioral factors, caloric balance, and follow-up support have the potential to induce it [15].

Sleeve gastrectomy is an effective surgical procedure for treating obesity. However, a significant percentage of patients suffer from weight regain in long-term follow-up. A better understanding of the causes of weight regain after bariatric surgery will tremendously advance this field and, in the future, give techniques for the identification, treatment, and prevention of obesity recurrence, which would considerably enhance bariatric surgery effectiveness. The purpose of the study is to evaluate the predictive effect of weight regain in the second year after sleeve gastrectomy on long-term bariatric surgery effectiveness.

## 2. Materials and Methods

A retrospective cohort study was performed using the database of routinely collected information about patients undergoing SG in the Department of General, Minimally Invasive and Elderly Surgery in Olsztyn. This database was collected through consultations. Patients undergoing SG were included in the study. The follow-up had to be for at least 5 years. Exclusion criteria were reoperation, failure to follow up, or poor short term weight results. Primary non-responders were determined according to bariatric criteria for weight loss success as > 50 percentage excess weight loss (%EWL) [16].

The following information was collected from the hospital database: patients’ sex, age, height, and weight from the day of surgery, length of hospital stay, operation time, domicile, and chronic diseases: T2D and AH. During the consultations, the patients completed postoperative questionnaires, giving their weight after one, two, and five years after the surgery, information on reoperation, and improvement in comorbidities. Each patient gave the appropriate consent to participate in the study, along with the consent to the Hospital’s Research Committee to conduct the study.

### 2.1. Outcomes Measurements

To determine the effectiveness of obesity treatment, three indicators were used: percentage of excess body mass index loss (%EBMIL), %EWL, and percentage total weight loss (%TWL) [17].

Patients were divided into two groups according to the change in body weight between the first and second years after the surgery. The first group (weight gainers, WG) included patients who started gaining weight in the second year after SG. The second group included patients who maintained or reduced their weight in the second year after surgery (weight maintainers, WM).

### 2.2. Statistical Analysis

Data were analyzed using descriptive statistics. The chi-square test was used to compare the proportions between WG and WM groups by sex, chronic diseases (T2D, AH), and domicile. Differences in mean age, initial body mass index (IBMI), mean %EBMIL and mean %EWL 5 years after surgery, operating time, length of hospital stay, and weight change five years after SG were assessed using the t-Student test for independent groups or Mann–Whitney U test. A *p*-value of 0 < 0.05 was considered significant. All calculations were preceded by Shapiro–Wilk and Levene tests. The analysis was performed using Statistica (data analysis software system), version 13, http://statistica.io (accessed on 10 August 2022) TIBCO Software Inc., Krakow, Poland (2017).

## 3. Results

**Figure 1:**
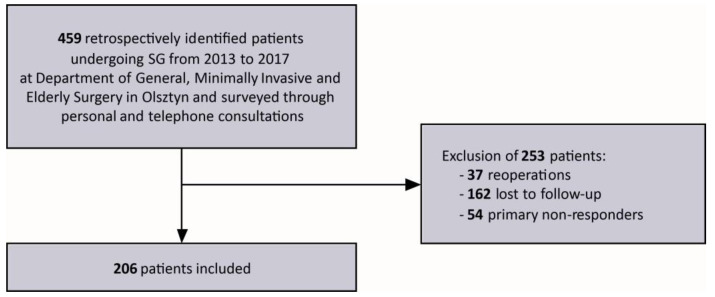
Flow chart of the study.

At the Department of General, Minimally Invasive and Elderly Surgery in Olsztyn, between 2013 and 2017, 459 patients underwent sleeve gastrectomy. The surgeries were performed by the same surgical team according to the standard technique [18]. We excluded 253 patients: 162 were not followed up with, 37 had been reoperated on, and 54 were primary non-responders. A total of 206 patients were included in the study (Figure 1). Follow-up rate was 64.71%.

The WG group consisted of sixty-nine patients (34%): fifty-two women and seventeen men (75%, 25%, respectively). The WM group consisted of 137 patients (66%): 112 women and 25 men (82%, 18%, respectively). There were no significant differences between the patient characteristics, (*p* > 0.05) (Table 1).

The mean %EBMIL, %EWL, and %TWL five years after SG for all patients were 90.23% (SD, 33.72%), 58.23% (SD, 21.41%), and 29.02 (SD, 10.84), respectively. The WM group had a mean %EBMIL change of 11.44% (SD, 24.73%), %EWL of 7.45% (SD, 15.83%), and %TWL of 3.74 (SD, 8.43). The WG group had a mean %EBMIL change of 34.96% (SD, 36.36%), %EWL of 22.78% (SD, 17.11%), and %TWL of 11.29 (SD, 8.68). The difference between the groups was statistically significant (*p* < 0.05) (Table 2).

The common criteria for bariatric success, 50% EBMIL, 50% EWL, and 25% TWL, showed significantly better results in WM compared to WG. Three-fourths of WM achieved bariatric success, but only 42% of WG achieved it, reaching the 50% EWL threshold (*p* < 0.05) (Table 2).

## 4. Discussion

According to our knowledge, this is the first study in bariatric surgery to assess the influence of weight gain in the second year after SG on the long-term effectiveness of the procedure. Patients were divided into two groups: WG and WM. Only 42% of WG met the 50% EWL criteria of bariatric success, compared to three-fourths of WM.

Different articles analyzed various models of weight loss after bariatric surgery. In a retrospective study, Lent et al. identified three weight loss trajectories after Roux-en-Y gastric bypass (RYGB) [11]. In two of them, 73% of respondents gained weight in the second year after the initial weight loss. In a study by Voorwinde et al., five weight loss trajectories have been established. In patients belonging to two trajectories, constituting a total of 49% of respondents, an increase in weight was noticed after its initial decrease in the first dozen months of treatment [12]. Courcoulas et al. assessed weight loss trajectories after RYGB and laparoscopic adjustable gastric banding (LAGB). For the RYGB six and LAGB seven, unique weight change trajectory patterns were found. Although patterns varied, the majority of patients followed trajectories in which they regained weight from 3 to 7 years after the surgery [13]. According to these studies, the important time period for weight regain to start is between the first and third years after bariatric surgery, which is consistent with our results, according to which, patients who regained weight in the second year after surgery had worse outcomes in terms of 50% EBMIL, 50% EWL and 25% TWL compared to those who maintained or reduced weight.

In addition to its many advantages, sleeve gastrectomy has several limitations. Weight loss may be insufficient in some patients. Some complications may be gender-specific, such as rhabdomyolysis, which is more common in men due to their initial greater muscle mass. The most common intraoperative complications are often undocumented or considered as nuisance bleeding, leakage, and gastric fistulae. Typical postoperative complications, although rare, include pulmonary embolism, hemorrhage, chest infections, abscess, incisional hernia, relaparoscopy for retained drain, anatomic leakage, wound infections, gastroesophageal reflux disease (GERD), and dumping. In addition, micronutrient deficiencies in vitamin B12, iron, calcium and vitamin D, thiamine, folate, and fat-soluble vitamins may develop during the first years after surgery. Moreover, and most significantly, weight loss may be unsatisfactory or weight may increase back with longer follow up [19].

Several predictive tools for weight loss after bariatric surgery have been developed [20,21]. The predictive calculator developed by Janik et al. to estimate the expected BMI one year after SG is one of them. It includes three factors that may influence weight loss: age, sex, and preoperative BMI [20]. Karpinska et al. created a retrospective analysis to evaluate the effectiveness of the available predictive models of potential body weight one year after RYGB and SG [21]. The study observed that all models overestimated the outcome and the accuracy of none of the models achieved an acceptable accuracy [21].

There are studies showing the impact of early weight loss on bariatric surgery effectiveness [22,23,24]. Manning et al. showed that early postoperative weight loss after SG can be used to identify patients who experience suboptimal weight loss after the procedure. In that retrospective study, 538 patients were measured 6 weeks and 6, 9, 12, 18, 24 months after the SG. Patients reached the lowest %WL in the interval between the 6th and 12th months. Additionally, %WL at 6 weeks, 3 and 6 months was significantly associated with maximal %WL. These findings are in line with our results. Our patients also reached the lowest %WL during the first year after the surgery. We see our study as an extension of Manning et al.’s research, as both our studies used weight change dynamics for further weight change prediction [22]. Tettero et al. revealed that patients in the lowest %TWL quartile group had less weight regain and were more likely to have lower weight loss up to 5 years in comparison to the Below Median, Above Median, and High %TWL quartile groups [23]. Yang et al., in a study conducted in two university hospitals, presented that preoperative age, WC, T2D, and %TWL at 1 or 3 months were associated with %TWL at 1 or 3 years after SG [24]. Although the prediction of long-term weight loss is multifactorial, our results suggest that weight gain in the second year after surgery may play an important role in predicting long-term sleeve gastrectomy effectiveness.

Concerning the estimation of potential weight regain, there are many studies analyzing the factors that can evoke it [15,25,26,27]. A systematic review of Yu et al. on weight regain after SG identified the following influencing factors: surgical/anatomic factors, increased residual gastric volume, gastric dilation, hormonal/metabolic imbalance, serotonin, behavioral/mood factors, energy consumption, and expenditure [15]. Different studies have also proposed different predictors for weight regain: ghrelin levels, follow-up control/support and lifestyle [25,26,27]. In our study, we did not collect data on or analyze the factors that were used to account for weight regain in the second year after bariatric surgery. However, it is a potential space to create new research results on these factors as well as ways to modify them.

Weight regain can be attributed to both patient- and surgery-related causes [28]. Moreover, we can distinguish between surgical factors and the specifics of the SG procedure for reasons related to the surgery. There are claims that the main disadvantage of the gastric sleeve is that it can enlarge over time. The postoperative BMI and the size of the gastric sleeve are directly associated. The initial size of the sleeve affects weight regain and insufficient weight loss. The size of the sleeve depends on bougie size, leaving fundal remnant, and size of antral remnant [29].

As is known from previous studies, long-term weight loss maintenance depends on the patient’s ability to adhere to a complex set of behaviors regarding dietary restrictions and lifestyle changes [30]. According to Ünal et al. weight regain is associated with depression, night eating, emotional eating, eating concerns, and the time elapsed after the surgery and being married. Meanwhile, knowledge about the amount of daily nutrients is needed to reduce the likelihood of it [31]. Bach et al., who assessed patients with obesity two weeks and 24 weeks after bariatric surgery using functional magnetic resonance, observed that the percentage of total weight loss (%TWL) was highest in candidates with high cue-induced food craving, the high-perceived feeling of hunger, and a low Yale food addiction scale sum score [32], While adherence to postoperative therapy and lifestyle changes has an immense effect on bariatric surgery effectiveness, objective measurement of those factors is a significant challenge [33]. We resigned from gathering such information during our retrospective study as the results reflected more of the patient’s personality and belief of one’s adherence to dietary factors and physical activity than the factual, indicator-based adherence.

Moreover, elderly patients with a prior history of AH who have undergone abdominal surgery, have depression/anxiety, comorbidities, or are unemployed despite weight loss are more likely to regain weight 2 years after bariatric surgery [34]. Assessing early postoperative weight loss or regain can help identify patients who show a poor response to surgery and enable the timely implementation of behavioral support that would potentially improve their long-term weight loss trend.

Compliance with 12-month postoperative follow-up is associated with improved weight loss [35]. Moreover, patients who routinely reported for follow-up visits for 3 years after surgery showed significantly greater weight loss compared to the group that stopped attending follow-up visits after a year or less [36]. Additionally, long follow-up studies show interluding relations between Quality of Life (QOL) and BMI changes. QOL reaches the highest level 1–2 years after the operation is assessed and then shows a gradual decline. Thus, follow-up should be extended even to 5 years or more, which could help maintain patients’ weight loss and keep QOL at the highest possible level [37,38].

Our intention is to promote awareness of the time after surgery when the patient starts regaining weight after reaching their lowest body weight. In a systematic review, Lauti et al. observed that weight regain after SG appeared in 5.7% after 2 years and in 75.6% after 6 years [14]. In a study by Felsenreich et al., 58.5% of patients regained weight over 10 years of follow up [39]. Capturing the point of deterioration in response to treatment could provide an opportunity for the timely introduction of additional measures and interventions that could help maintain the target body weight.

Recurrence of comorbidities and other serious consequences for patients as well as the healthcare system make weight recidivism a key public health concern. The complex, multifaceted, and sometimes overlapping causal etiology contributes to weight increase. A planned and methodical strategy is therefore crucial to managing weight regain [40]. Additionally, it is essential to develop and implement screening tools for a long-term follow-up that could be easily used by physicians to identify patients who require a comprehensive evaluation and additional support to maintain the surgical outcomes [41].

There are some limitations in the study. The study design—retrospective cohort study—has some limitations: selection bias, because the sample of participants may not be representative; recall bias, as patients may have some difficulty recalling events from the last 5 years; covariates, because the group was not controlled after surgery, so it was difficult to establish the relationship between the variables; and incomplete data as the follow-up rate was around 65%. A significant number of patients were failed to be followed up (35%), so we could only speculate that their results were as expected. Primary non-responders and reoperated patients are also likely to be a subgroup of patients for whom SG has generally failed to produce the desired results. However, reoperation patients could not be analyzed due to incomparable results in the standard timeline. Another factor that increases the risk of bias is the limited sample. A total of 206 respondents were included. Compared to studies conducted on large datasets, our study may be less credible. Body mass indicators count the entire body weight, not dividing it into individual tissues. For example, a significant amount of muscle tissue might be treated as adipose tissue. In addition, registry-based studies carry an inherent risk of misleading factors that have not been adjusted. The reason for weight regain could have been the occurrence of additional diseases, the patient’s burden of socioeconomic causes, pregnancy, or many other causes. Patients were not asked about the potential cause of weight regain, and men and women were not directly comparable. The limitations of this study also include the bias in selecting unoperated participants. Reoperated patients were not included in the study. The most common reason for reoperation was the treatment of gastroesophageal reflux disease, whose influence on bariatric surgery effectiveness is yet to be determined. Although these patients are potentially part of the WG group, they could not be compared because their weight loss curve was quite different from patients who had not undergone other surgeries.

## 5. Conclusions

Weight regain in the second year after SG may be a good factor for long-term bariatric surgery effectiveness prognosis. Implementation of long-term follow-up is essential for the early detection of patients who are more likely to fail and for the establishment of additional therapeutic strategies to prevent weight regain or even reoperation. However, future research is needed.

## Figures and Tables

**Table 1:** Characteristics of groups. Participant characteristics for: all patients, patients who started gaining weight in the second year after the surgery (WG) and patients who maintained or reduced weight (WM). * *p*-value refers to differences between WM and WG groups.

	All	WG Group	WM Group	*p*-Value *
**N**	206	69 (33.5%)	137 (66.5%)	
Gender				0.283
Female (%)	164 (80%)	52 (75%)	112 (82%)	
Male (%)	42 (20%)	17 (25%)	25 (18%)	
Mean age (±SD) [years]	39.03 (10.20)	39.25 (10.52)	38.25 (10.08)	0.877
Mean IBMI (±SD) [kg/m^2^]	43.38 (5.99)	42.97 (5.37)	43.58 (6.28)	0.627
T2D				0.714
Yes (%)	36 (17%)	13 (19%)	23 (17%)	
No (%)	170 (83%)	56 (81%)	114 (83%)	
AH				0.870
Yes (%)	79 (38%)	42 (61%)	85 (62%)	
No (%)	127 (62%)	27 (39%)	52 (38%)	
Mean operation time (±SD) [min]	71.93 (26.07)	73.84 (26.18)	70.90 (26.05)	0.355
Mean length of hospital stay (±SD) [days]	3.30 (1.88)	3.26 (1.21)	3.32 (2.16)	0.902
Domicile size (number of inhabitants)				0.222
<25,000 (%)	93 (45%)	37 (54%)	56 (41%)	
25,000–100,000 (%)	32 (16%)	9 (13%)	23 (17%)	
>100,000 (%)	81 (39%)	23 (33%)	58 (42%)	

**Table 2:** Postoperative results for: all patients, patients who started gaining weight in the second year after the surgery (WG), and patients who maintained or reduced weight (WM). * *p*-value refers to differences between WM and WG groups.

	All	WG Group	WM Group	*p*-Value *
Mean %EBMIL 5 years after SG (±SD)	90.23 (33.72)	77.18 (34.88)	96.80 (31.23)	<0.005
Mean %EWL 5 years after SG (±SD)	58.23 (21.41)	50.00 (22.74)	62.38 (19.51)	<0.005
Mean %TWL 5 years after SG (±SD)	29.02 (10.84)	24.62 (11.07)	31.24 (10.05)	<0.005
Mean change in %EBMIL between 1st and 5th year after SG (±SD)	19.32 (27.54)	34.96 (26.26)	11.44 (24.73)	<0.005
Mean change in %EWL between 1st and 5th year after SG (±SD)	12.58 (17.78)	22.78 (17.11)	7.45 (15.83)	<0.005
Mean change in %TWL between 1st and 5th year after SG (±SD)	6.27 (9.22)	11.29 (8.68)	3.74 (8.43)	<0.005
≥50% EBMIL 5 years after SG				0.003
Yes (%)	181 (88%)	54 (78%)	127 (93%)	
No (%)	25 (12%)	15 (22%)	10 (7%)	
≥50% EWL 5 years after SG				<0.005
Yes (%)	138 (67%)	37 (54%)	106 (77%)	
No (%)	68 (33%)	32 (46%)	31 (23%)	
≥25% TWL 5 years after SG				<0.005
Yes (%)	132 (64%)	29 (42%)	103 (75%)	
No (%)	74 (36%)	40 (58%)	34 (25%)	

## Data Availability

Not applicable.
